# FLI1 regulates radiotherapy resistance in nasopharyngeal carcinoma through TIE1-mediated PI3K/AKT signaling pathway

**DOI:** 10.1186/s12967-023-03986-y

**Published:** 2023-02-22

**Authors:** Enni Chen, Jiajia Huang, Miao Chen, Jiawei Wu, Puyun Ouyang, Xiaonan Wang, Dingbo Shi, Zhiqiao Liu, Wancui Zhu, Haohui Sun, Shanshan Yang, Baoyu Zhang, Wuguo Deng, Huijuan Qiu, Fangyun Xie

**Affiliations:** State Key Laboratory of Oncology in South China, Guangdong Key Laboratory of Nasopharyngeal Carcinoma Diagnosis and Therapy, Collaborative Innovation Center of Cancer Medicine, Sun Yat-Sen University Cancer Center, Guangzhou, 510060 China

**Keywords:** FLI1, TIE1, Nasopharyngeal carcinoma (NPC), Radiotherapy resistance

## Abstract

**Background:**

Radiotherapy resistance is the main cause of treatment failure in nasopharyngeal carcinoma (NPC), which leads to poor prognosis. It is urgent to elucidate the molecular mechanisms underlying radiotherapy resistance.

**Methods:**

RNA-seq analysis was applied to five paired progressive disease (PD) and complete response (CR) NPC tissues. Loss-and gain-of-function assays were used for oncogenic function of FLI1 both in vitro and in vivo. RNA-seq analysis, ChIP assays and dual luciferase reporter assays were performed to explore the interaction between FLI1 and TIE1. Gene expression with clinical information from tissue microarray of NPC were analyzed for associations between FLI1/TIE1 expression and NPC prognosis.

**Results:**

FLI1 is a potential radiosensitivity regulator which was dramatically overexpressed in the patients with PD to radiotherapy compared to those with CR. FLI1 induced radiotherapy resistance and enhanced the ability of DNA damage repair in vitro, and promoted radiotherapy resistance in vivo. Mechanistic investigations showed that FLI1 upregulated the transcription of TIE1 by binding to its promoter, thus activated the PI3K/AKT signaling pathway. A decrease in TIE1 expression restored radiosensitivity of NPC cells. Furthermore, NPC patients with high levels of FLI1 and TIE1 were correlated with poor prognosis.

**Conclusion:**

Our study has revealed that FLI1 regulates radiotherapy resistance of NPC through TIE1-mediated PI3K/AKT signaling pathway, suggesting that targeting the FLI1/TIE1 signaling pathway could be a potential therapeutic strategy to enhance the efficacy of radiotherapy in NPC.

**Supplementary Information:**

The online version contains supplementary material available at 10.1186/s12967-023-03986-y.

## Background

Nasopharyngeal carcinoma (NPC), as an epithelial carcinoma originating from the mucosa of the nasopharynx, is particularly common in East and Southeast Asia [1]. Because of its complex anatomical location and high radiosensitivity, intensity-modulated radiotherapy is the main treatment for NPC [2]. Despite the continuous improvement of the clinical treatment methods, 19–29% of the patients still have recurrence and distant metastasis after radiotherapy, which significantly reduces the survival time and life quality [3–6]. Radiotherapy resistance is the main cause of the treatment failure of NPC [7, 8]. Therefore, it is highly necessary to explore the biomarker and molecular mechanism of radiotherapy resistance of NPC.

FLI1 (friend leukemia virus integration 1) is a common proviral integration site related to erythroleukemia induced by Friend Murine Leukemia Virus (F-MuLV) [9]. FLI1 belongs to the E26 transformation-specific gene (ETS) family that has a DNA binding domain responsible for DNA recognition on target promoters. As a transcription factor, FLI1 plays an important role in the development of hematopoietic stem cells, vasculogenesis and angiogenesis. However, abnormal expression of FLI1 induces auto-immune diseases, as well as different kinds of human cancers [10–14]. In breast cancer, transcriptional upregulation of CDHI and VIM by FLI1 contributes to invasion and migration [12], and upregulation of BCL2 inhibits apoptosis [15]. In diffuse large B-cell lymphoma, FLI1-mediates regulation of chemokine receptors (CXCR5, CXCR4 and CXCR7) participates in the neoplastic process [14]. The t (11; 22)(q24; q12) chromosomal translocation produces EWS-FLI, a transcriptional activator that mediates oncogenic transformation [16]. Additionally, EWS-FLI1 has been extensively studied in Ewing’s sarcoma [17–19]. It has been demonstrated that EWS-FLI1 acts in a positive feedback loop to maintain the expression of PARP1, thus promoting DNA damage repair [20]. Consistently, other members of the ETS family, such as ERG, ETS1 and ETS2, are also associated with repair of DNA damage [21–23]. Pathways involved in DNA damage repair are critical for the treatment outcome of radiotherapy [24]. In addition, overexpression of FLI1 in glioblastoma cells promotes resistance to both radiation and temozolomide [25]. Previously, we have demonstrated the expression of FLI1 is an independent prognostic factor for NPC patients [26]. Nevertheless, the role of FLI1 in radiosensitivity of NPC remains largely unknown.

TIE1 (tyrosine kinase with immunoglobulin like and EGF like domains 1) is a tyrosine kinase receptor first isolated from an erythroleukemia cell line [27]. Previous studies suggest that TIE1, which expresses primarily in endothelial cells, plays a very important part in blood and lymphatic vascular development [28]. TIE1 is critical to embryonic development, and ablation of TIE1 causes hemorrhages, edema, and destruction of microvascular integrity, leading to mid- to late-gestation embryonic lethality [29]. However, activation of TIE1 in endothelial cells upregulates several pro-inflammatory genes, thereby inducing endothelial inflammatory response [30]. TIE1 deficiency in mouse tumor model decreases tumor angiogenesis and enhances vascular normalization, leading to increased tumor necrosis, which ultimately results in delayed tumor growth at later stage [31]. Besides, TIE1 expresses in various epithelial cancers, such as gastric cancer [32], colon cancer [33], thyroid cancer [34] and breast cancer [35, 36]. In ovarian cancer, TIE1 promotes XPC-dependent nucleotide excision repair (NER) to render platinum reagents resistance [37]. Moreover, TIE1 plays a critical role in ovarian cancer cell proliferation and growth by modulating the PI3K/AKT signaling pathway [38].

In this study, we identified the role of FLI1 in regulating radiotherapy resistance in NPC and verified FLI1 as a transcription activator of TIE1. We further demonstrated that FLI1 activated the PI3K/AKT signaling pathway by upregulating TIE1, thereby affecting NPC radiotherapy sensitivity. Our study indicates the FLI1/TIE1 signaling axis may be a new mechanism of NPC radiotherapy resistance and a potential therapeutic target for NPC treatment.

## Results

### High FLI1 expression is associated with radioresistance and poor prognosis in NPC patients

**Fig. 1:**
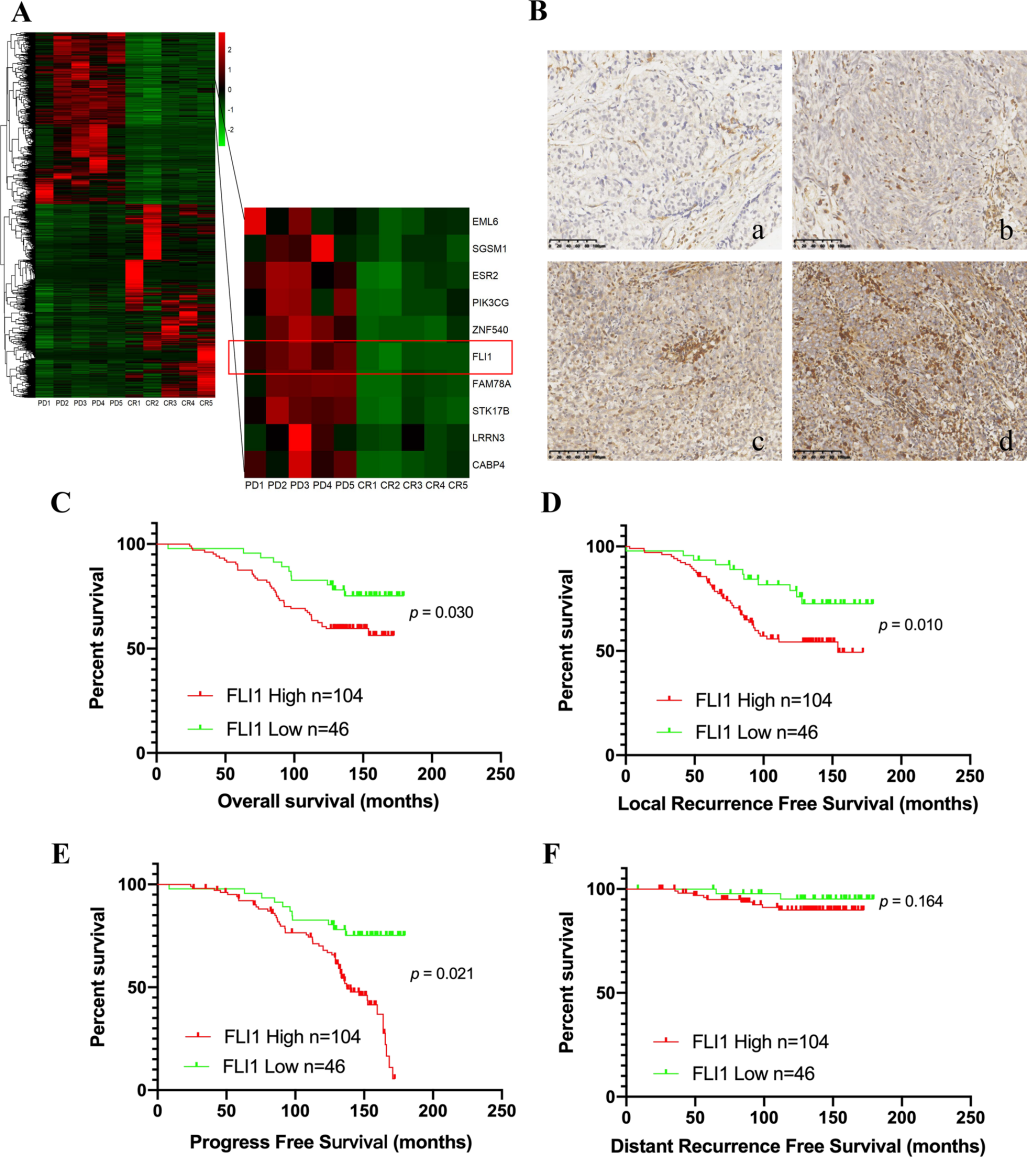
High FLI1 levels are associated with radioresistance and poor prognosis in NPC patients. **A** Heatmap profiling of the gene expression in radiosensitive tissues (n = 5) and radioresistant tissues (n = 5), as is analyzed by RNA-seq. **B** Representative images of immunohistochemical (IHC) staining of FLI1 protein expression in 150 NPC tissues (a, no brown particle staining; b, light brown particles; c, moderate brown particles; d, dark brown particles). Scale bars = 100 μm. **C–F** Kaplan–Meier analysis of the overall survival **C**, local recurrence free survival **D**, progress free survival **E** and distant recurrence free survival **F** of 150 NPC patients based on FLI1 expression. The *p* values were analyzed by log-rank test

To explore the main genes that modulate radiation resistance in NPC, we collected five NPC biopsy tissues that exhibited complete response (CR) to radiotherapy at 6 months and the other five NPC biopsy tissues that exhibited progressive disease (PD) and then performed RNA sequencing (RNA-Seq) analysis. KEGG enrichment analysis showed that the DNA replication pathway was enriched (Additional file 1: Fig. S1). Differential gene expression analysis showed that FLI1 was dramatically overexpressed in PD tissues compared to CR tissues (Fig. 1A). FLI1 protein expression was detected in human NPC tissue microarrays by IHC staining (Fig. 1B). Pearson chi-square analysis showed that patients in advanced stages had higher FLI1 expression levels (Additional file 2: Table S1). In addition, Kaplan-Meier survival analysis showed that NPC patients with high FLI1 expression levels had a remarkably shorter overall survival (OS, *p* = 0.030), local recurrence free survival (LRFS, *p* = 0.010) and progress free survival (PFS, *p* = 0.021) (Fig. 1C–E). However, the distant recurrence free survival (DRFS) between patients with high and low FLI1 levels had no significant difference (*p* = 0.164) (Fig. 1F). According to these results, we speculated that high FLI1 levels in NPC tissues may result in resistance to radiotherapy.

### FLI1 facilitates NPC cell survival after irradiation (IR) in vitro

**Fig. 2:**
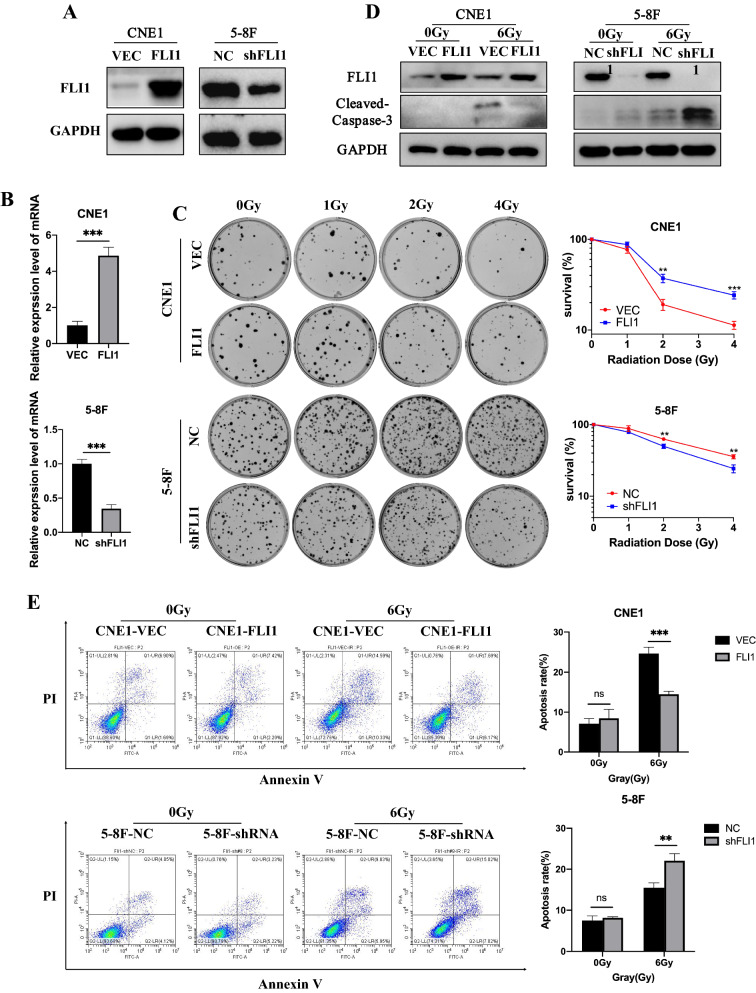
FLI1 facilitates NPC cell survival after irradiation (IR) in vitro. **A–B** Western blot **A** and RT-qPCR **B** analysis of FLI1 protein and mRNA level in CNE1 cells with FLI1 overexpression and 5-8F cells with FLI1 knockdown (VEC, control lentiviral vector; FLI1, FLI1 overexpression lentiviral vector; NC, negative control shRNA; shFLI1, FLI1-specific shRNA). GAPDH was included as a loading control. **C** Cells were seeded at the density of 200, 400, 800 and 1000 cells for 0 Gy, 1 Gy, 2 Gy and 4 Gy IR dose. Colony formation assays and survival fraction curve analysis were employed to assess cell survival at 10–14 days after exposure to indicated IR dose. **D**–**E** Western blot **D** and Annexin V/PI double-staining **E** assays were performed to evaluate the effects of FLI1 on apoptosis 48 h after cells treated with or without IR. Data in B, C and E are presented as mean ± SD (n = 3). **p < 0.01, ***p < 0.001, ****p < 0.0001; *ns* not significant (Student’s t-test). Source data are provided as a Source Data file

To explore the effect of FLI1 on radiosensitivity of NPC, we stably overexpressed FLI1 in CNE1 and SUNE1 cells which had lower FLI1 levels and were more radiosensitive, and knocked down FLI1 in 5-8F and 6-10B cell lines which had higher FLI1 levels and were more radioresistant (Fig. 2A, B and Additional file 1: Fig. S2A–E). We performed clonogenic assays after IR treatment, and found that FLI1 overexpression enhanced clonogenic survival in CNE1 and SUNE1 cells upon IR, while FLI1 knockdown obviously impeded clonogenic survival in 5-8F and 6-10B cells upon IR (Fig. 2C and Additional file 1: Fig. S2F). The western blot analysis revealed that the level of the radiation-induced apoptosis-related protein, cleaved caspase-3, was reduced in FLI1-overexpressing CNE1 cells, but elevated in FLI1-silenced 5-8F cells, compared with the respective controls (Fig. 2D). In addition, apoptosis assays showed that FLI1 overexpression significantly decreased radiation-induced apoptosis in CNE1 and SUNE1 cells, whereas FLI1 knockdown increased radiation-induced apoptosis in 5-8F and 6-10B cells (Fig. 2E and Additional file 1: Fig. S2G).

### FLI1 induces radioresistance by facilitating DNA double-strand breaks (DSBs) repair in NPC cells

**Fig. 3:**
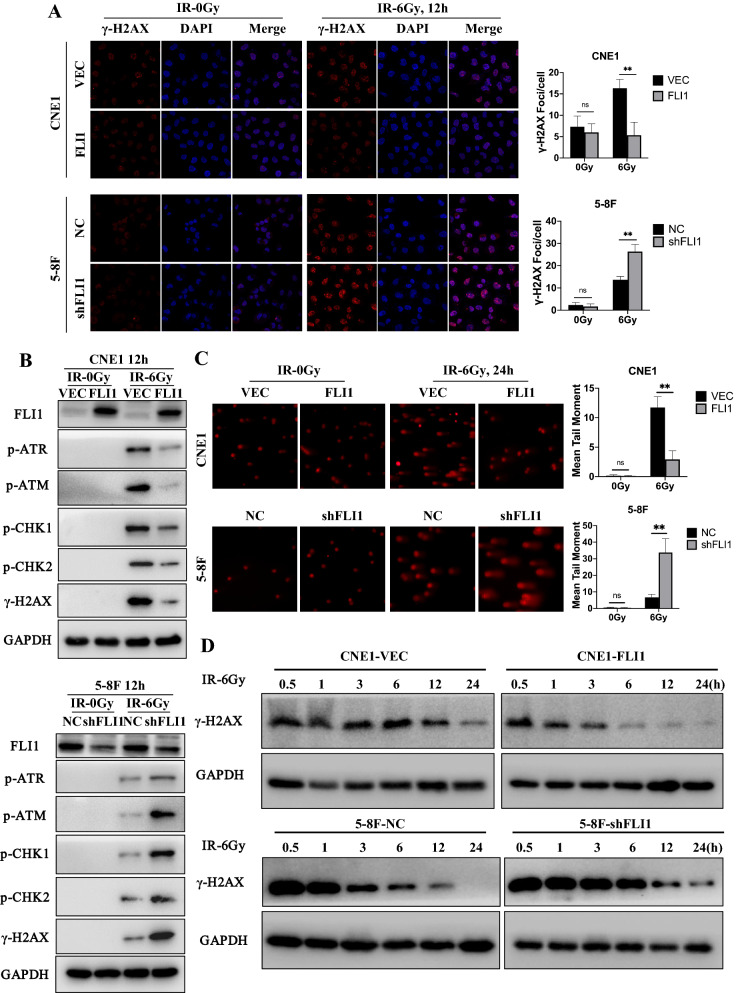
FLI1 induces radioresistance by facilitating DNA double-strand breaks (DSBs) repair in NPC cells. **A** Immunofluorescence assays to examine the effect of FLI1 on γ-H2AX foci formation in the indicated CNE1 and 5-8F cells with or without IR. **B** Western blot assays of γ-H2AX, p-ATR, p-ATM, pCHK1 and p-CHK2 protein levels in the indicated CNE1 and 5-8F cells with or without IR. **C** Comet images and quantitative analysis of tail moments for DNA damage in the indicated CNE1 and 5-8F cells with or without IR treatment. **D** Western blot analysis of the time course of changes in γ-H2AX levels in NPC cells with 6 Gy IR exposure. Data in **A** and **C** are presented as mean ± SD (n = 3). **p < 0.01; *ns* not significant (Student’s t-test). Source data are provided as a Source Data file

Phosphorylation of histone H2AX (γ-H2AX) is considered as one of the earliest markers in response to DSBs caused by ionizing radiation [39]. Western blot assays showed lower γ-H2AX protein levels in 5-8F and 6-10B cells than in CNE1 and SUNE1 cells, which was consistent with the baseline sensitivity of the cell lines in Additional file 1: Fig. S2C (Additional file 1: Fig. S3). Therefore, we investigated the impact of FLI1 on γ-H2AX levels in NPC cells after radiation by western blot and immunofluorescence assays. The results showed fewer γ-H2AX foci and lower γ-H2AX protein levels in the CNE1-OE cells than in CNE1-VEC cells at 12 h after radiation exposure. By contrast, more γ-H2AX foci and higher γ-H2AX protein levels were observed in 5-8F-shFLI1 cells than in 5-8F-NC control cells at 12 h post radiation (Fig. 3A, B). In addition, as shown in Fig. 3B, we detected the protein levels of other key mediators of DNA repair and replication. The levels of phosphorylated ATR (p-ATR), phosphorylated ATM (p-ATM), phosphorylated CHK1 (p-CHK1) and phosphorylated CHK2 (p-CHK2) in CNE1-OE cells and 5-8F-NC cells were significantly lower than in CNE1-VEC control cells and 5-8F-shFLI1 cells respectively. We then performed comet assays at 24 h after radiation to detect DNA damage levels. We found that the levels of DNA damage indicated by mean tail moment in CNE1-OE cells was significantly lower than in CNE1-VEC cells after IR, while 5-8F-shFLI1 cells exhibited higher DNA damage levels than control cells (Fig. 3C).

Furthermore, to prove whether FLI1 influenced radiation-induced DNA damage repair, we detected γ-H2AX protein levels at different time points after radiation by western blot assays. As shown in Fig. 3D, the γ-H2AX levels decreased more rapidly in CNE1-OE cells and 5-8F-NC cells than in CNE1-VEC control cells and 5-8F-shFLI1 cells respectively, indicating that FLI1 facilitated DNA lesion repair in NPC cells.

### FLI1 binds to TIE1 promoter and activates TIE1 transcription in NPC cells

**Fig. 4:**
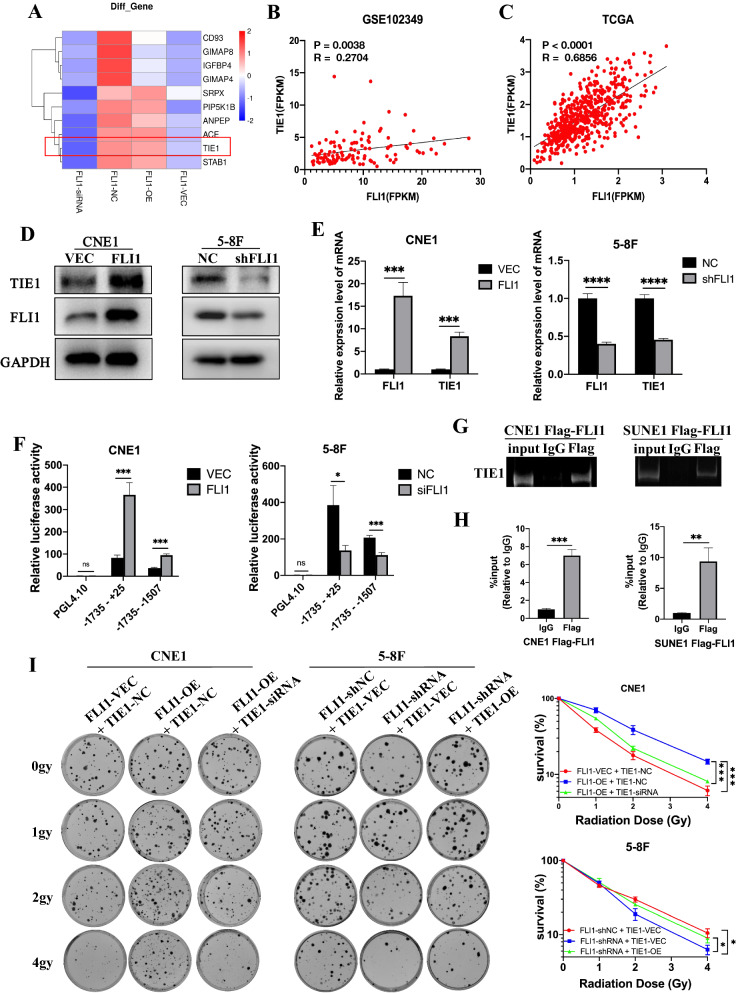
FLI1 binds to TIE1 promoter and activates TIE1 transcription in NPC cells. **A** Heatmap showing differential gene expression between CNE1-OE cells and CNE1-VEC cells, 5-8F-shRNA cells and 5-8F-NC cells, as is analyzed by RNA-seq. **B**–**C** Correlation analysis between FLI1 and TIE1 in GEO database **B** and TCGA database **C**. **D**–**E** Western blot **C** and RT-qPCR **D** analysis of TIE1 protein and mRNA level in FLI1 overexpression and knockdown NPC cells. **F** Dual-luciferase reporter assays were used to evaluate TIE1 promoter activity in NPC cells transiently transfected with control vector (VEC), FLI1 overexpression plasmid (FLI1), negative control siRNA (NC) and FLI1-specific siRNA (siFLI1). **G**–**H** CNE1 and SUNE1 cells were transiently transfected with vector (VEC) and Flag-FLI1 overexpression plasmid (Flag-FLI1). ChIP-PCR **G** and ChIP-qPCR **H** assays of TIE1 promoter region were conducted with Flag or control IgG antibody in CNE1 Flag-FLI1 and SUNE1 Flag-FLI1 cells. **I** CNE1-VEC and CNE1-FLI1 cells were transiently transfected with siTIE1 or control siRNA. 5-8F-NC and 5-8F-shFLI1 cells were transiently transfected with TIE1 or empty vector plasmids. Cells were seeded at the density of 200, 400, 800 and 1000 cells for 0 Gy, 1 Gy, 2 Gy and 4 Gy IR dose. Colony formation assays and survival fraction curve analysis were employed to assess cell survival at 10–14 days after exposure to indicated IR dose. Data in **E**, **F**, **H** and **I** are presented as mean ± SD (n = 3). *p < 0.05, **p < 0.01, ***p < 0.001, ****p < 0.0001; ns, not significant (Student’s t-test). Source data are provided as a Source Data file

To identify the underlying mechanism of FLI1 in regulating NPC radioresistance, we performed RNA-Seq analysis to search for differentially expressed genes between CNE1-OE and CNE1-VEC cells, as well as 5-8F-shFLI1 and 5-8F-NC cells respectively. The up-regulated genes induced by FLI1 overexpression were intersected with down-regulated genes caused by FLI1 knockdown, and the differentially expressed genes were identified. Heatmap showed the top 10 differentially expressed genes and TIE1 was among the candidates (Fig. 4A). The data from the Gene Expression Omnibus (GEO) NPC database and the Cancer Genome Atlas (TCGA) head and neck squamous cell carcinoma (HNSCC) datasets showed that the levels of FLI1 and TIE1 are highly positively correlated (Fig. 4B, C). Since FLI1 regulates the expression of its target genes by binding to their promoter regions, we explored whether it directly modulated the expression of TIE1, which has been reported to promote XPC-dependent nucleotide excision repair (NER) and chemotherapy resistance [38]. hTFtarget ChIP-seq database also indicated that FLI1 might transcriptionally regulate TIE1 by binding to its promoter region (Additional file 3: hTFtarget ChIP-seq data).

Firstly, we detected the regulation of TIE1 mRNA and protein levels by FLI1 in NPC cells. Western blot and RT-qPCR assays demonstrated that the protein and mRNA levels of TIE1 were increased when FLI1 was up-regulated, but were significantly decreased when FLI1 was knocked down (Fig. 4D, E and Additional file 1: Fig. S4A, B). To identify the binding site of FLI1 on the TIE1 promoter, we performed luciferase reporter assays with two different TIE1 promoter-driven luciferase reporter segments. The results showed that FLI1 overexpression promotes TIE1 promoter activity corresponding to the fragments from − 1735 to + 25 and from − 1735 to -1507, whereas FLI1 knockdown suppressed the TIE1 promoter activity (Fig. 4F and Additional file 1: Fig. S4C). ChIP assays also verified that FLI1 bound to the TIE1 promoter region from − 1735 to − 1507 (Fig. 4G, H).

In addition, to validate that the FLI1 regulated NPC radioresistance depends on its transcription of TIE1, rescue experiments were performed in NPC cells. As shown in Fig. 4I, increased clonogenic survival induced by FLI1 overexpression was partly offset by TIE1 knockdown in CNE1 cells. In contrast, TIE1 overexpression reversed the FLI1 knockdown-mediated decreased clonogenic survival in 5-8F cells, confirming that FLI1 controlled NPC radiosensitivity through TIE1.

### FLI1 regulates TIE1-mediated PI3K/AKT signaling pathway in NPC cells

**Fig. 5:**
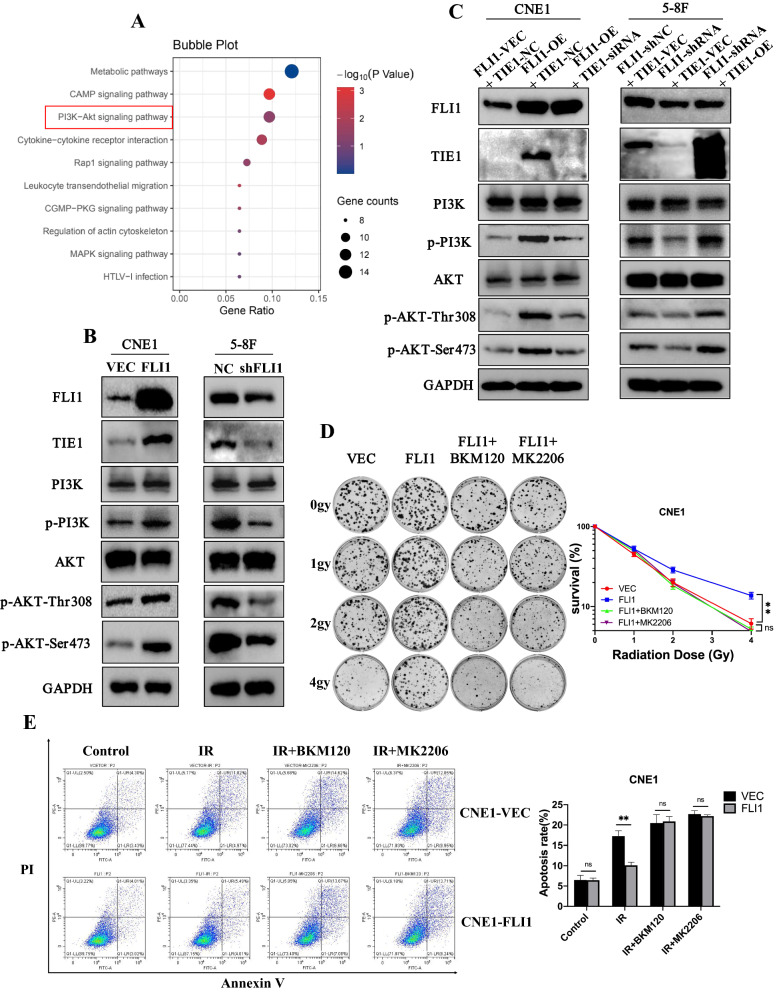
FLI1 regulates TIE1-mediated PI3K/AKT signaling pathway in NPC cells. **A** KEGG pathway analysis of genes regulated by FLI1 in CNE1 and 5-8F cells. PI3K/AKT pathway was among the significant pathways. **B** Western blot analysis of TIE1, PI3K, p-PI3K, AKT, p-AKT (Thr308) and p-AKT (Ser473) in CNE1 cells with FLI1 overexpression and 5-8F cells with FLI1 knockdown. **C** CNE1-VEC and CNE1-FLI1 cells were transiently transfected with siTIE1 or control siRNA. 5-8F-NC and 5-8F-shFLI1 cells were transiently transfected with TIE1 or empty vector plasmids. Western blot assays were performed to assess the protein level of TIE1, PI3K, p-PI3K, AKT, p-AKT (Thr308) and p-AKT (Ser473). **D**–**E** CNE1-VEC and CNE1-FLI1 cells were treated with IR, a PI3K inhibitor BKM120 (3 μM) and an AKT inhibitor MK2206 (3 μM). Colony formation assays and survival fraction curve analysis **D** were employed to assess cell survival at 10–14 days after exposure to indicated IR dose. Cell apoptosis **E** was determined by Annexin V/PI double-staining assays at 48 h after indicated treatment. Data in D and E are presented as mean ± SD (n = 3). **p < 0.01; ns, not significant (Student’s t-test). Source data are provided as a Source Data file

Accumulating evidence suggests that PI3K/AKT signaling pathway is associated with radioresistance of cancers [40–42]. TIE1 has been reported to be related to the PI3K/AKT signaling pathway in ovarian cancer [38]. KEGG pathway analysis indicated that FLI1 regulated PI3K/AKT signaling pathway (Fig. 5A). Thus, we hypothesized that FLI1 activated PI3K/AKT signaling pathway through regulating TIE1 expression. We first assessed the influence of FLI1 on phosphorylation levels of PI3K and AKT. The results showed that up-regulation of FLI1 increased the phosphorylation levels of PI3K and AKT, while knockdown of FLI1 showed the opposite results (Fig. 5B and Additional file 1: Fig. S5A). To determine whether FLI1 regulated PI3K/AKT signaling pathway through TIE1, we performed rescue experiments in NPC cells. In CNE1 cells, the increased phosphorylation levels of PI3K and AKT-mediated by FLI1 were decreased after TIE1 knockdown, while TIE1 overexpression reversed the levels of phosphorylation PI3K and AKT in 5-8F-shFLI1 cells (Fig. 5C). In addition, the promotive effects of FLI1 overexpression on clonogenic survival were reversed by PI3K and AKT inhibitors (Fig. 5D). In contrast, the suppressive effects on CNE1 cell apoptosis and γ-H2AX protein levels induced by FLI1 overexpression were reversed by the inhibitors (Fig. 5E and Additional file 1: Fig. S5B). Overall, these results suggest that the TIE1 mediated PI3K/AKT signaling pathway is a functional target of FLI1 that promotes the radioresistance in NPC cells.

### FLI1 induces radioresistance in the NPC xenograft mouse model

**Fig. 6:**
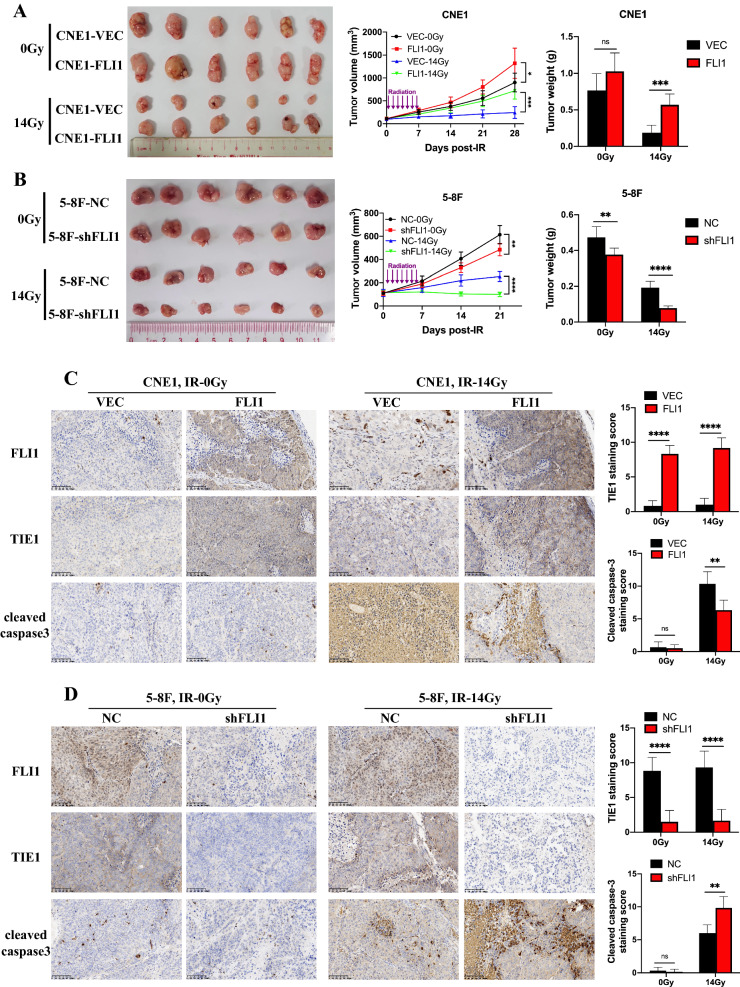
FLI1 induces radioresistance in the NPC xenograft mouse model. **A**–**B** Assessment of FLI1 levels on radiotherapy efficacy in NPC xenografts. FLI1-overexpressing (FLI1) and empty vector (VEC)-transfected CNE1 cells **A** or FLI1-knockdown (shFLI1) and negative control (NC) 5-8F cells **B** were implanted into BALB/c nude mice, which were exposed to IR or not. Tumor volume and weight of the excised tumors were measured (left, excised tumors; middle, tumor volume; right, tumor weight). **C**–**D** Representative images of IHC staining and IHC scores for FLI1, TIE1 and cleaved caspase-3 in the excised tumors. Scale bars = 100 μm. **p < 0.01, ****p < 0.0001; *ns* not significant (Student’s t-test). Source data are provided as a Source Data file

To determine whether FLI1 promotes the radioresistance of NPC cells in vivo, we generated nude mouse xenograft model. We established subcutaneous NPC xenografts by injecting CNE1-FLI1 and 5-8F-shRNA cells and their respective control cells. After that, we treated the mice with IR (2 Gy/day for 7 consecutive days). As shown in Fig. 6A, compared with the VECTOR group, the FLI1 group exhibited increased NPC tumor growth in terms of size, volume, and weight in response to radiation. On the other hands, knockdown of FLI1 markedly suppressed tumor growth after radiation (Fig. 6B). Furthermore, IHC staining assays showed stronger TIE1 and weaker cleaved caspase-3 staining in the tumors of the CNE1-FLI1 group compared with those of the CNE1-VEC group after IR treatment, and the opposite effects were found in the tumors of the 5-8F-shFLI1 group compared with those of the control group after IR exposure (Fig. 6C, D). These data suggest that FLI1 regulates TIE1 expression, thus inhibiting radiation-induced apoptosis to confer NPC cell radioresistance in vivo.

### FLI1 expression is positively correlated with TIE1 expression and high FLI1-TIE1 levels predict poor clinical outcomes in NPC patients

**Fig. 7:**
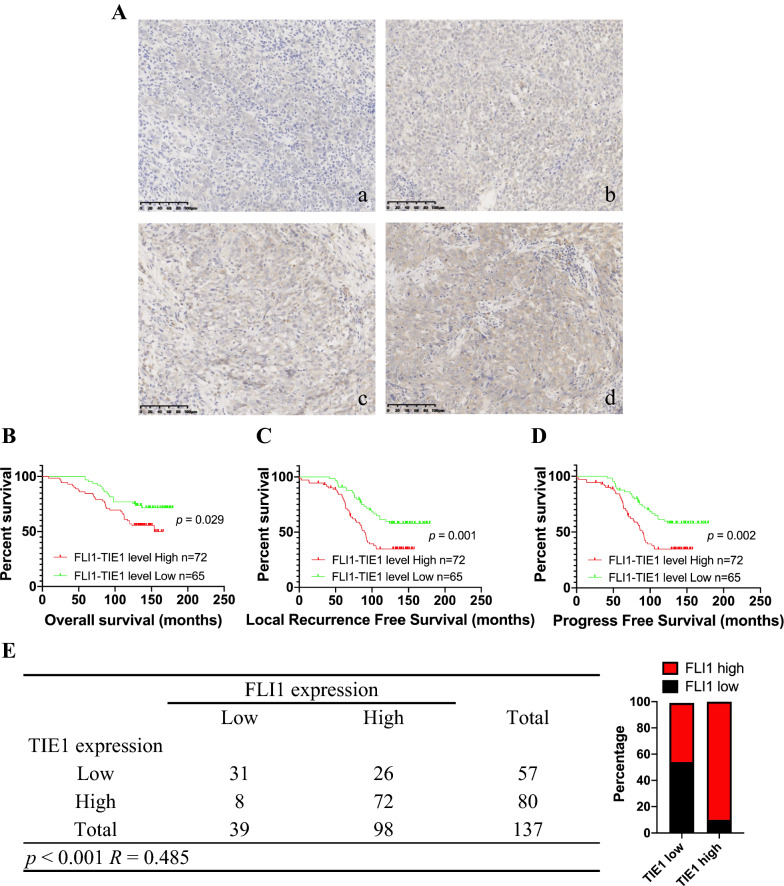
FLI1 expression is positively correlated with TIE1 expression and high FLI1-TIE1 levels predict poor clinical outcomes in NPC patients. **A** Representative images of IHC staining of TIE1 protein expression in 137 NPC tissues (a, no brown particle staining; b, light brown particles; c, moderate brown particles; d, dark brown particles). Scale bars = 100 μm. **B**–**D** Kaplan–Meier analysis of the overall survival **B**, local recurrence free survival **C** and progress free survival **D** of 137 NPC patients based on TIE1 expression. The *p* values were analyzed by log-rank test. **E** The correlation analysis between FLI1 expression and TIE1 expression. Pearson chi-square analysis was used to determine the correlation

To clarify association between FLI1/TIE1 expression and their roles in the prediction of clinical prognosis, we used IHC staining assays to detect the expression of FLI1 and TIE1 in 137 NPC patients using tissue microarrays (Fig. 7A and Fig. 2B). Kaplan–Meier analysis showed that NPC patients with high FLI1-TIE1 levels had a shorter OS (*p* = 0.020), LRFS (*p* = 0.002) and PFS (*p* = 0.003) (Fig. 7B–D). In addition, multivariate COX-regression analysis showed that FLI1-TIE1 level was an independent prognostic factor (Additional file 2: Tables S2, S3, S4). Among the patients, 52.6% (72 of 137) exhibited both high expression of FLI1 and TIE1 expression, while 22.6% (31 of 137) exhibited both low expression of FLI1 and TIE1. Correlation analysis showed that FLI1 expression was positively correlated with TIE1 expression (R = 0.485, *p* < 0.001) (Fig. 7E).

## Discussion

Although the cure rate of early-stage NPC remains high, most NPC patients are diagnosed with advanced stages due to its hidden growth and non-specific symptoms in early stages. For patients in advanced stages, radiotherapy combined with chemotherapy has become the main treatment strategy [1, 43]. The therapeutic effectiveness in NPC has been enhanced by the application of IMRT combined with concurrent chemotherapy, as well as the accuracy of NPC staging and target volume delineation improved by PET/CT and MRI respectively [44]. However, a portion of patients still suffer from recurrence and distant metastasis afterwards [3–6]. Radioresistance is commonly recognized as the crucial obstacle for the cure of NPC [45, 46]. Therefore, it is urgent to elucidate the molecular mechanism of radiotherapy resistance and identify potential therapeutic targets. In this study, we have revealed the FLI1/TIE1 signaling axis as a new target for tackling NPC radioresistance.

Studies have reported that high expression of FLI1 promotes the generation and progression of a variety of solid tumors, such as Ewing's sarcoma, breast cancer, melanoma, prostate cancer and glioma [10, 47]. Previously, we have identified FLI1 as an independent prognostic factor significantly associated with the survival in NPC patients [26]. It has been widely acknowledged that ionizing radiation induces DNA DSBs to kill tumor cells. However, DNA DSBs also activate complicated and highly regulated DNA damage response and repair (DRR) signaling, which leads to radioresistance in cancer cells [24, 48]. The members of the ETS family are often involved in DNA damage repair. For instance, EWS-FLI1 and ERG interact with Parp1 and DNA-PKcs respectively, thus mediating the DRR signaling pathway [20, 21]. On the other hand, some ETS genes can regulate the expression of DNA repair elements in cancer. ETS1 is reported to up-regulate PARP1 in Ewing sarcoma and ovarian cancer [22, 49], while ETS1 and ETS2 repress BRCA1 in breast cancer [50, 51]. Besides, ETS2 interacts with mutant p53 to increase the expression of TDP2, which promotes resistance to etoposide-induced DNA damage [52]. So far, the role of FLI1 in DNA damage has rarely been reported. FLI1 is shown to promote glioblastoma radioresistance by regulating HSBP1 [25], and its expression is associated with radiation resistance in oral squamous cell carcinoma [53]. However, the relationship between FLI1 and radiotherapy resistance in NPC and the underlying mechanisms remain unknown. Here, we firstly analyzed the differentially expressed genes between radioresistant and radiosensitive NPC groups, and found that FLI1 was noticeably elevated in radioresistant tissues. We next demonstrated that FLI1 facilitates NPC cell survival and reduces cell apoptosis after IR in vitro and vivo. As γH2AX serves as a platform for the recruitment of other DRR factors and amplification of signaling pathways [24, 39], we explored the influence of FLI1 on γ-H2AX levels after radiation. Our results indicated that FLI1 knockdown significantly impaired the DNA damage repair activity induced by radiation in NPC cells.

To clarify the potential mechanism by which FLI1 regulates radioresistance in NPC, we performed RNA-seq analysis and identified TIE1 as a candidate target through heatmap analysis. ETS1 and ETS2 are shown to transactivate TIE1 promoter [54]. Previous studies have demonstrated that TIE1, which expresses primarily in endothelial cells, is associated with vascular pathologies, including tumor angiogenesis and atherosclerosis [31, 55–57]. It is known that TIE1 is upregulated in tumor vessels, thus contributing to tumor progression, but its roles in tumor cells have been rarely explored [31, 32, 34, 35, 58–62]. TIE1 is found to mediate platinum resistance in ovarian cancer cells through nucleotide excision repair [37]. In non-small cell lung carcinoma cells, it contributes to cisplatin resistance through promoting cell stemness [63]. Additionally, it regulates PI3K/AKT signaling, which is critical in cancer radiotherapy and chemotherapy resistance [38, 42]. Our results from ChIP and luciferase reporter assays showed that FLI1 activated the transcription of TIE1 by binding to its promoter region from -1735 to -1507. In line with this, the rescue experiments confirmed that the FLI1-mediated radioresistance was TIE1 dependent.

The PI3K/AKT signaling pathway plays indispensable roles in cancer cell proliferation, differentiation and apoptosis, leading to cancer progression [64, 65]. Accumulating evidence indicates that PI3K/AKT signaling promotes radiotherapy resistance in tumor cells through regulating DRR processes. Activation of AKT triggers the accumulation of DNA-PKcs at the site of DSBs, promoting NHEJ DNA-DSB repair [66]. Upon activation of PI3K/AKT pathway, mTORC1 increases the synthesis of homologous recombination (HR) repair proteins, such as RAD50, RAD51 and BRCA1 [67]. FLI1 has been reported to influence PI3K/AKT signaling in breast cancer, and EWS-FLI1 fusion gene enhances PI3K/AKT/mTOR signaling in Ewing sarcoma [12, 68]. It is well known that PI3K is necessary for the activation of AKT by phosphorylation on Threonine^308^ (T308) and Serine^473^ (S473) [69, 70]. We confirmed that FLI1 regulated TIE1-mediated PI3K/AKT signaling pathway to affect radiosensitivity of NPC cells. Further study is needed to identify whether FLI1 regulates TIE1 by cooperating or antagonizing with other factors.

In summary, these data demonstrated that FLI1 transcriptionally upregulated TIE1 expression by binding to its promoter, and thus activated PI3K/AKT signaling pathway, leading to NPC cell radioresistance. Furthermore, our findings revealed that NPC patients with high FLI1-TIE1 levels were related to poor prognosis, suggesting that targeting FLI1/TIE1 could be a potential therapeutic strategy to enhance the efficacy of NPC radiotherapy in the future.

## Materials and methods

### Clinical specimens

We collected 10 freshly frozen NPC biopsy tissues from patients who did not receive any anti-tumor therapy in Sun Yat-Sen University Cancer Center (Guangzhou, China). After a standard anti-tumor therapy, half of the patients exhibited complete response (CR) to radiotherapy at 6 months, which means sensitive to radiotherapy, while the others exhibited progressive disease (PD), which means resistant to the therapy. Besides, NPC tissue microarrays including 150 cases were purchased from Shanghai Outdo Biotech company (TFNas0-01). The tumor tissues were all took from patients without anticancer therapies before biopsy.

### Cell culture and reagents

Human NPC cell lines (CNE1, CNE2, SUNE1, 5-8F, 6-10B and C666) were cultured in RPMI-1640 (Invitrogen, Carlsbad, CA, USA) supplemented with 10% foetal bovine serum (FBS, Gibco). The NPC cell lines were generously provided by Professor Jun Ma (Sun Yat-sen University Cancer Center, China) and had been authenticated. HEK293T cells purchased from the American Type Tissue Culture Collection (ATCC) were cultured in DMEM (Invitrogen) with 10% FBS. All cell lines were cultured in a humidified atmosphere with 5% CO2 at 37 °C.

Buparlisib (BKM120; S2247) and MK-2206 (Selleck; S1078) were purchased from Selleck Chemicals.

### Plasmid construction and transfection

The Flag-tagged FLI1 and TIE1 were cloned into the pSIN-EF2-puro vector to obtain the overexpression plasmids. FLI1 shRNA sequence was cloned into the PLKO.1-puro vector to obtain PLKO.1-shFLI1 plasmid. The different fragments of the TIE1 promoter region were synthesised and cloned into the pGL4-basic luciferase reporter vector. The siRNAs targeting FLI1 and TIE1 were purchased from RiboBio (Guangzhou, GD, China). The sequences of siRNA and shRNA are listed in Additional file 2: Table S5.

For transient transfection, the plasmids and siRNAs were transfected with Lipofectamine@3000 transfection reagent (Invitrogen) into indicated cells according to the manufacturer’s protocols. For stable transfection, HEK293T cells were transfected with plasmids and the virus supernatant of it was collected at 48 h. The supernatant containing virus was then infected NPC cells with polybrene. The transfected cells were selected with puromycin (2 μg/ml) at least 1 week.

### Clonogenic assay

Single-cell suspensions were seeded into 6-well plates at the density of 200, 400, 800 and 1000 cells per well. The cells were treated with IR at the doses of 0, 1, 2 and 4 Gy until cell adherence and then cultured for 10–14 days. After staining with crystal violet, colonies containing more than 50 cells were counted.

### Apoptosis assay

Apoptosis was detected based on FACS analysis with an Annexin V/PI double-staining assay. Cells were collected after treated with or without IR, and then stained with the Annexin V/PI apoptosis kit (BD Biosciences, NJ, USA). The apoptosis rate was analyzed using flow cytometry.

### RNA extraction and RT-qPCR assay

Cell lines RNA was extracted with RaPure Total RNA Micro Kit (Magen, Guangzhou, GD, China) according to the manufacturer’s protocols. Complimentary DNA was produced using HiScript II Q RT SuperMix for qPCR kit (Vazyme, Piscataway, NJ, USA). qPCR assays were conducted using ChamQ SYBR Green qPCR Master Mix (Vazyme) and CFX96 Touch sequence detection system (Bio-Rad). The primer sequences used are listed in Additional file 2: Table S5.

### Western blot assay

Cell lines were lysed with RIPA lysis buffer to obtain total protein. Total protein was separated by SDS-PAGE and transferred to PVDF membranes (Merck Millipore, Billerica, MA, USA). The membranes were then blocked in 5% skim milk and incubated with primary antibodies at 4 °C overnight. After incubated with HRP-linked secondary antibodies (anti-mouse or anti-rabbit) at room temperature for 1 h, the protein bands were detected using the ChemiDoc MP Imaging System (Bio-Rad). The antibodies used are listed in Additional file 2: Table S6.

### Confocal immunofluorescence assay

Cells were fixed in 4% paraformaldehyde for 15 min, permeabilized in 0.5% Triton-X for 5 min, blocked in 1% BSA-PBS for 30 min and then incubated in primary antibodies at 4 °C overnight. The primary antibody was diluted in 1% BSA. The secondary antibody was then added to the samples and incubated for 1 h in the dark room. Finally, the cells were stained with 4′,6-diamidino-2-phenylindole (DAPI; sigma, St. Louis, MO) and anti-fade reagent. We used the confocal scanning microscope (LSM880 with Fast Airyscan, ZEISS) to capture the images. The antibodies used are listed in Additional file 2: Table S6.

### Comet assay

Cells were treated with IR (6 Gy) and harvested at 12 h after IR. We used a comet assay kit (KeyGen Biotech, Nanjing, JS, China) according to the manufacturer’s instructions. After stained with propidium iodide (PI), comet images were captured by fluorescence microscopy, and CaspLab-Comet Assay Software was used to analyze the tail moments.

### Dual luciferase reporter assay

CNE1, SUNE1, 5-8F and 6-10B cell lines, which transiently transfected with the plasmids or siRNA against FLI1 for 24 h, were seeded into 24-well plates and transfected with luciferase reporter plasmids. The cells were co-transfected with Renilla luciferase to normalize the transfection efficiency. After 48 h transfection, the relative luciferase activity was exanimated using Dual Luciferase Reporter Assay Kit (Promega, Madison, WI, USA) in accordance with the manufacturer’s instruction.

### ChIP assay

We used SimpleChIP@ Enzymatic Chromatin IP kit (Cell Signaling Technology; 9002S) to conduct ChIP assay. About 1 × 10^7^ cells were cross-linked by 1% formaldehyde and quenched with 125 mmol/L glycine. Next, we lysed and sonicated the cells to yield 150–900 bp DNA fragments. Chromatin was immunoprecipitated with Flag antibody or IgG. DNA was then isolated to quantify by qPCR or PCR. The primer sequence is listed in Additional file 2: Table S5. The antibody used is listed in Additional file 2: Table S6.

### High-throughput mRNA-sequence and data analysis

Five paired of radiosensitive and radioresistant NPC tissues were collected to perform RNA-seq, which was carried by Sinotech Genomics Co., LTD (Shanghai, China). Besides, total RNA of 5-8F cells transfected with siRNA against FLI1, and CNE1 cells transfected with FLI1 overexpression plasmid was isolated to perform RNA-seq. Thresholds of P < 0.05 and FDR ≤ 0.25 were used to select differentially expressed genes. The significant genes were subjected to Kyoto Encyclopedia of Genes and Genomes (KEGG) pathway analysis.

### Animals and treatment

BALB/c nude mice (4–5 weeks old; female; 15–18 g) were purchased from Gempharmatech Co., Ltd,. For the xenograft models, 1 × 10^6^ NPC cells were injected into the the right flank of mice. Once the tumor became palpable (100–150 mm^3^), the mice were treated with IR at 2 Gy per day for 7 consecutive days. Tumor volume was calculated as tumor volume = 0.52 × width^2^ × length. We measured the tumor sizes three times a week. The mice were sacrificed 3 or 4 weeks after IR, and the tumors were paraffin-embedded for pathological analysis.

### Immunohistochemistry (IHC) assay

IHC staining was performed on 4 μm sections. The slides were incubated with primary antibodies at 4 °C overnight and stained with 3,3′-diaminobenzidine (DAB; Dako, Santa Clara, CA, USA) for color development. The IHC results were evaluated by two different experienced pathologists based on the immunoreactive score (IRS) system. The intensity of staining was divided into four scores: 0 (no brown particle staining), 1 (light brown particles), 2 (moderate brown particles) and 3 (dark brown particles). The percentage of positive tumor cells was classified into four scores: 1 (< 10% positive cells), 2 (10–40% positive cells), 3 (40–70% positive cells) and 4 (> 70% positive cells). The two scores were multiplied to obtain the IRS scores (ranged from 0 to 12), which were used to determine high (score ≥ 3) or low (score < 3) levels. The antibodies used are listed in Additional file 2: Table S6.

### Database

TIE1 promoter sequence was obtained from UCSC database [https://genome.ucsc.edu]. We used GEO database (GSE102349) [https://www.ncbi.nlm.nih.gov/gds] and TCGA data in xena ucsc database [http://xena.ucsc.edu] to analyze FLI1 and TIE1 expression in NPC and HNSCC. The ChIP-seq data of FLI1 were obtained from hTFtarget database [https://ngdc.cncb.ac.cn/databasecommons/database/id/6946].

### Statistics and reproducibility

All data were analyzed and graphed using SPSS (version 26.0, Chicago, IL, USA) and GraphPad Prism (version 8.0, San Diego, CA, USA). Data were presented as mean ± standard deviation (SD) of three independent trials. Differences between groups were analyzed by unpaired two-side Student’s t-test and *p* < 0.05 was considered statistically significant. The Kaplan-Meier method was used to conduct the survival curve, and the log-rank test was performed to compare the differences between the groups. The correlation between expression of FLI1 and TIE1 was conducted using Pearson Chi-Square. Western blots were conducted three times independently with similar results.

## Supplementary Information

**Additional file 1:** **Figure S1.** KEGG pathway analysis of differentially expressed genes between CR and PD group. DNA replication pathway was among the significant pathways. **Figure S2.** **(A-B) **Western blot **(A) **and RT-qPCR **(B) **analysis of FLI1 expression in NPC cells. **(C) **Cells were seeded at the density of 200, 400, 800 and 1000 cells for 0Gy, 1Gy, 2Gy and 4Gy IR dose. Colony formation assays were performed and survival fraction curve analysis were employed to assess cell survival at 10-14 days after exposure to indicated IR dose. **(D-E)** Western blot **(D)** and RT-qPCR **(E)** analysis of FLI1 expression in SUNE1 cells with FLI1 overexpression and 6-10B cells with FLI1 knockdown. **(F) **Colony formation assays and survival fraction curve analysis were employed to assess cell survival at 10-14 days after exposure to indicated IR dose. **(G)** Annexin V/PI double-staining assays were performed to evaluate the effects of FLI1 on apoptosis 48h after cells treated with or without IR. Data in C, E ,F and G are presented as mean ± SD (n=3). *p < 0.05, **p < 0.01, ***p < 0.001, ****p < 0.0001; ns, not significant (Student's t-test). Source data are provided as a Source Data file. **Figure S3.** Western blot of γ-H2AX protein levels in indicated NPC cells with or without IR. **Figure S4.** **(A-B) **Western blot **(A)** and RT-qPCR **(B)** analysis of TIE1 protein and mRNA level in FLI1 overexpression and knockdown NPC cells.** (C) **Dual-luciferase reporter assays were used to evaluate TIE1 promoter activity in NPC cells transiently transfected with control vector (VEC), FLI1 overexpression plasmid (FLI1), negative control siRNA (NC) and FLI1-specific siRNA (siFLI1). **Figure S5.** **(A)** Western blot analysis of TIE1, PI3K, p-PI3K, AKT, p-AKT (Thr308) and p-AKT (Ser473) in SUNE1 cells with FLI1 overexpression and 6-10B cells with FLI1 knockdown. **(B) **CNE1-VEC and CNE1-FLI1 cells were treated with IR, a PI3K inhibitor BKM120 (3μM) and an AKT inhibitor MK2206 (3μM). Western blot analysis was performed to detect the protein levels of γ-H2AX.

**Additional file 2:** **Table S1.** Correlation between FLI1 and clinical characteristics in NPC patients. **Table S2.** Multivariate analysis of prognostic factors for OS in NPC patients. **Table S3.** Multivariate analysis of prognostic factors for LRFS in NPC patients. **Table S4.** Multivariate analysis of prognostic factors for PFS in NPC patients. **Table S5.** List of primers used in this study. **Table S6.** List of antibodies used in this study.

**Additional file 3:** hTFtarget ChIP-seq data: The ChIP-seq data of FLI1.

## Data Availability

The data that support the findings of this study are available from the corresponding author upon reasonable request.
