# Supplementary figures and images for: FLI1 regulates radiotherapy resistance in nasopharyngeal carcinoma through TIE1-mediated PI3K/AKT signaling pathway
Source: J Transl Med. 2023 Feb 22;21:134. doi: 10.1186/s12967-023-03986-y (PMC9945741; doi:10.1186/s12967-023-03986-y)

Figure S1

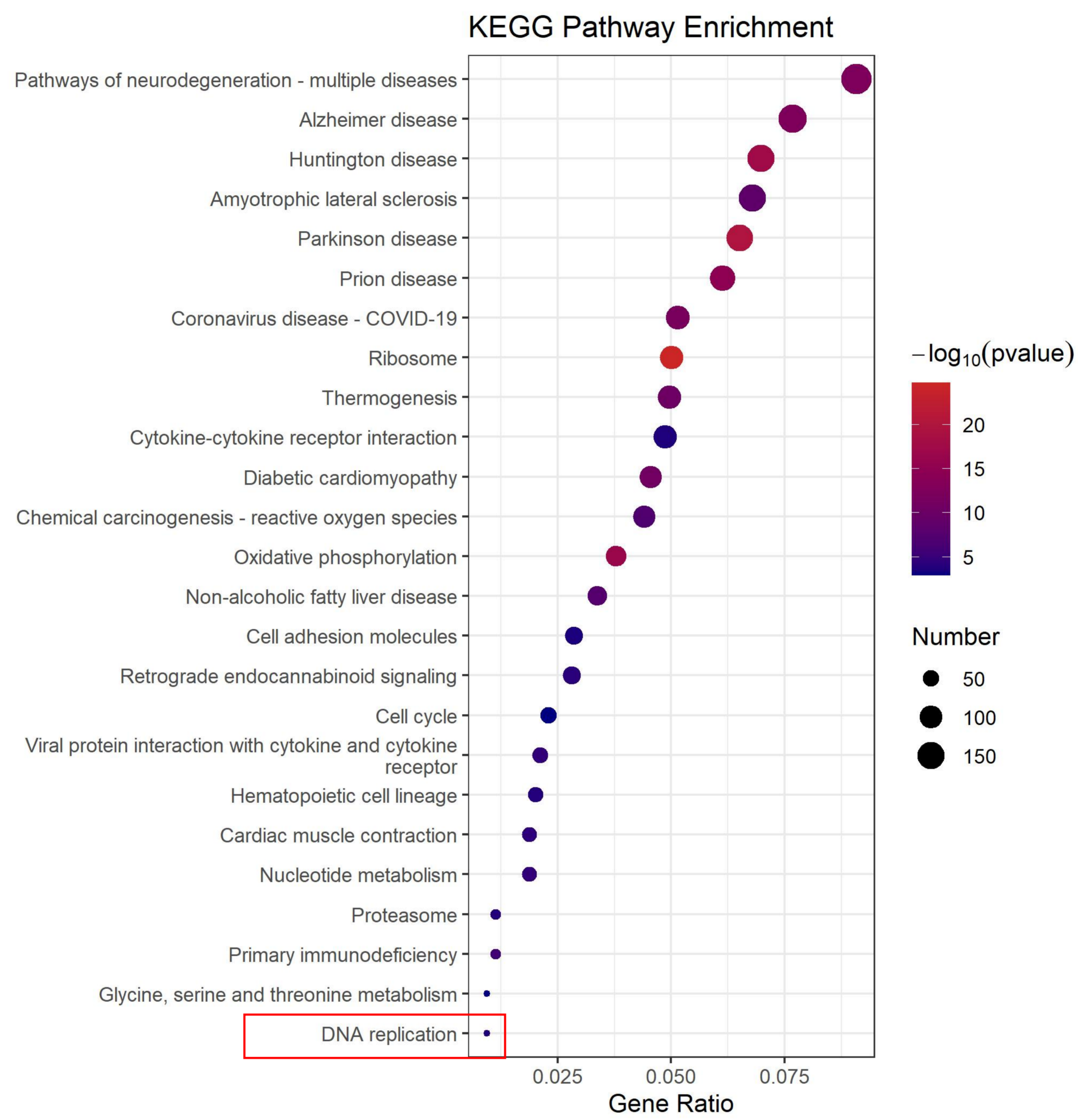

Figure S2

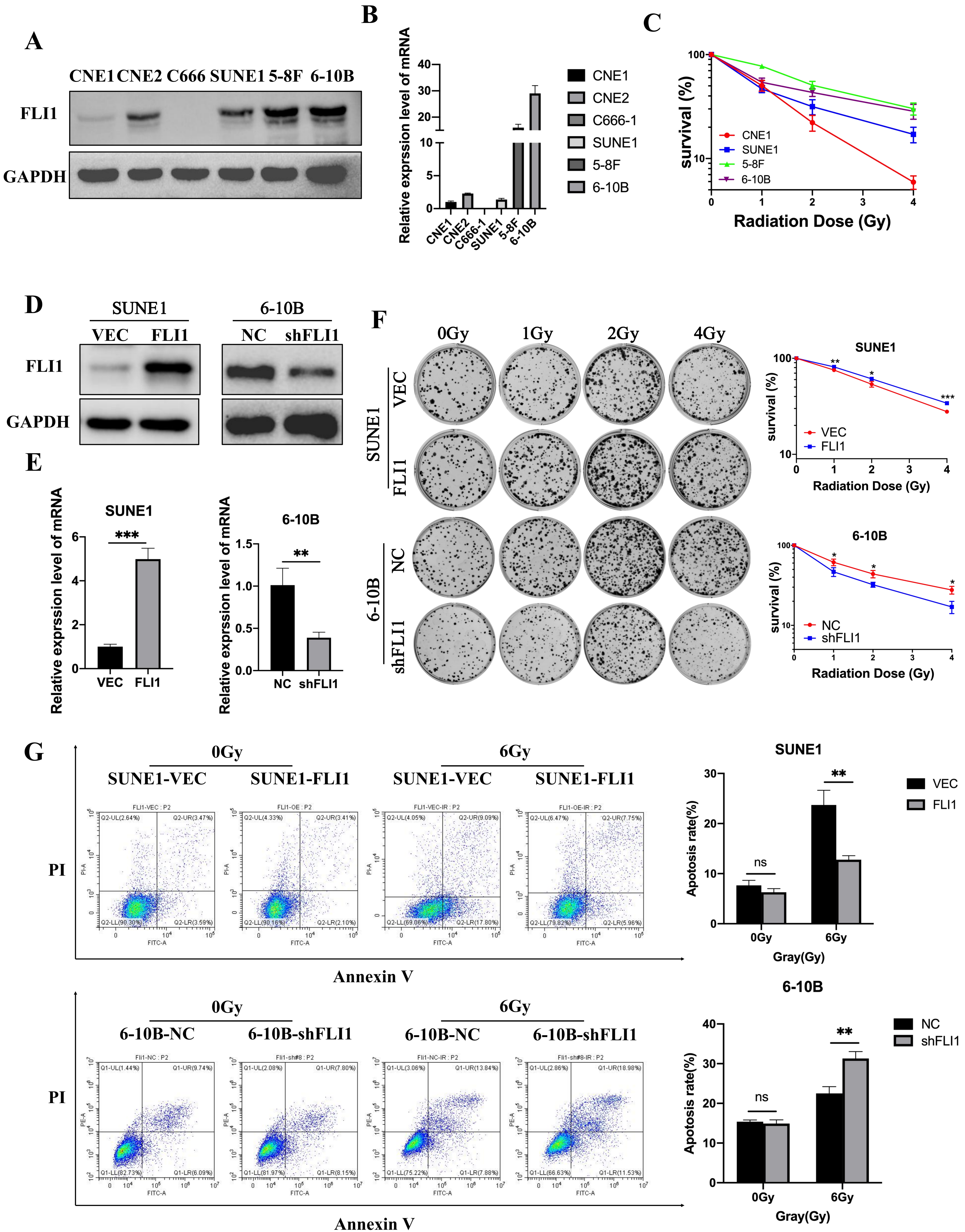

Figure S3

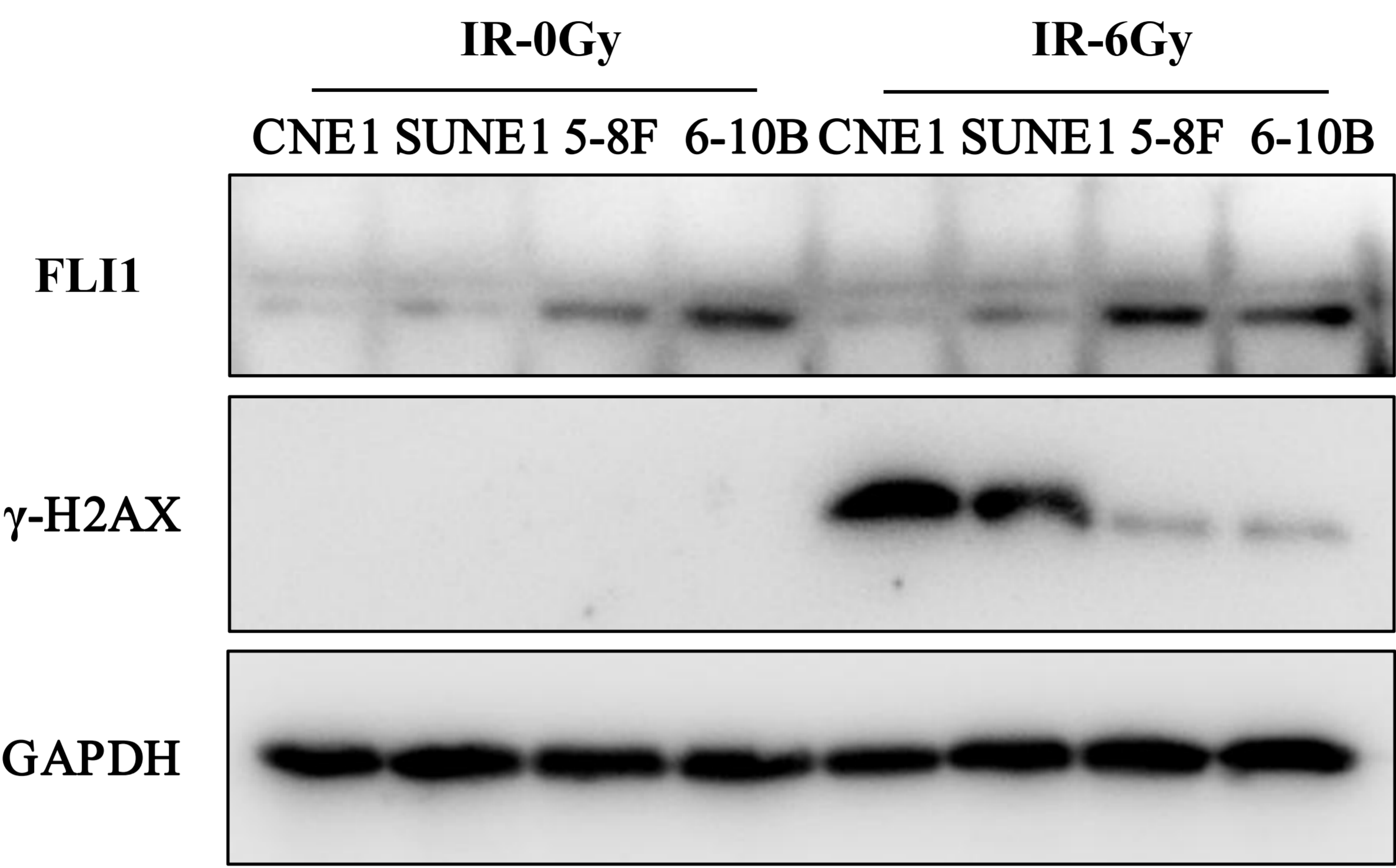

Figure S4

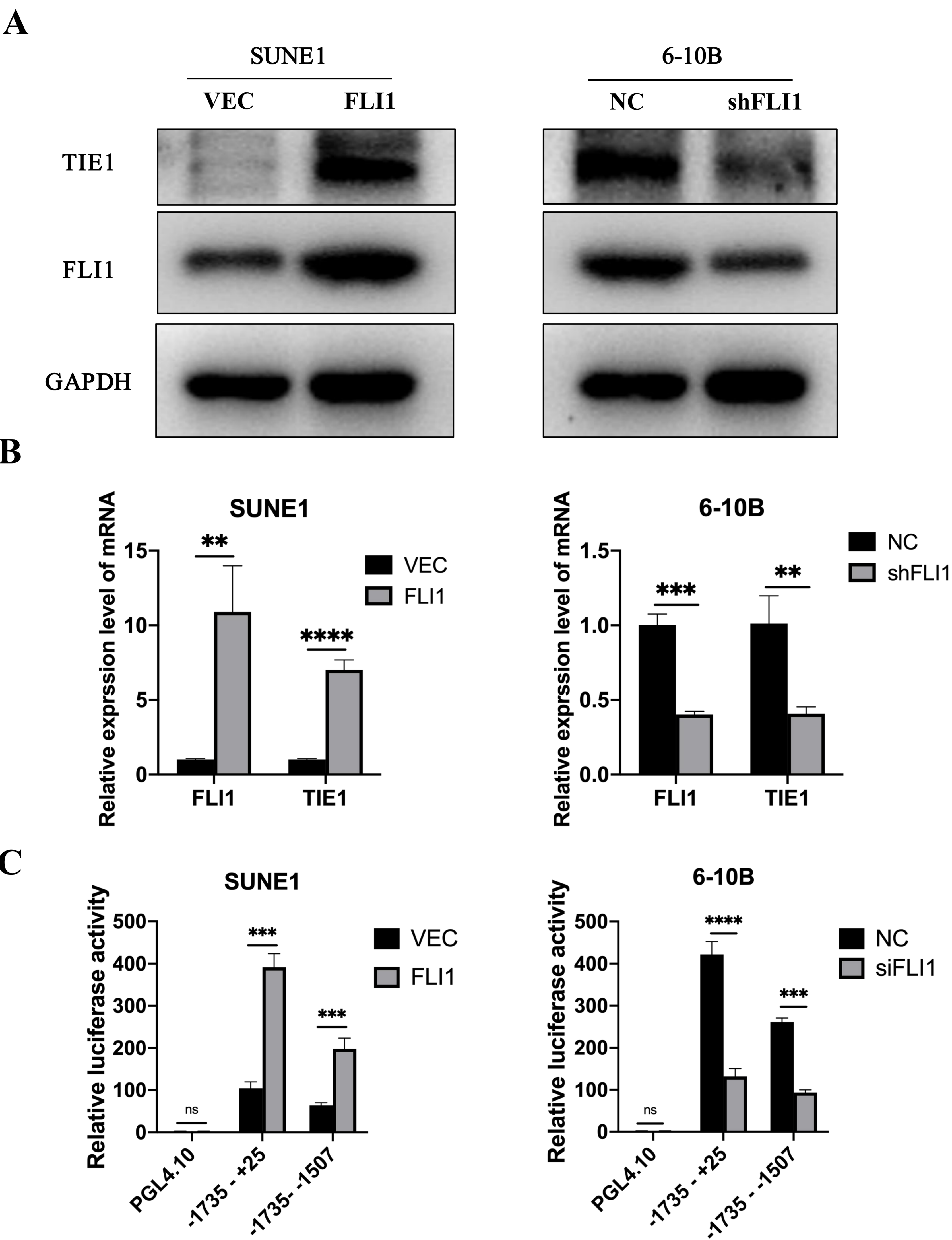

Figure S5

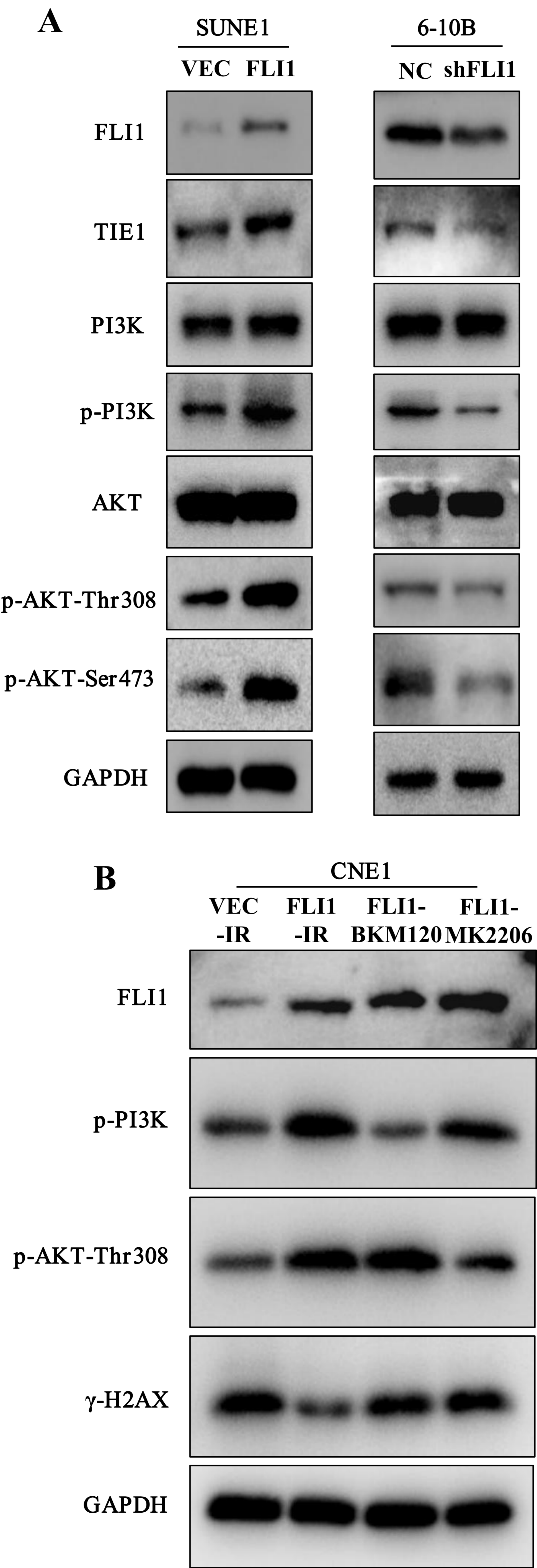

Supplement: Supplementary file 1 — Additional file 1: Figure S1. KEGG pathway analysis of differentially expressed genes between CR and PD group. DNA replication pathway was among the significant pathways. Figure S2. (A-B) Western blot (A) and RT-qPCR (B) analysis of FLI1 expression in NPC cells. (C) Cells were seeded at the density of 200, 400, 800 and 1000 cells for 0Gy, 1Gy, 2Gy and 4Gy IR dose. Colony formation assays were performed and survival fraction curve analysis were employed to assess cell survival at 10-14 days after exposure to indicated IR dose. (D-E) Western blot (D) and RT-qPCR (E) analysis of FLI1 expression in SUNE1 cells with FLI1 overexpression and 6-10B cells with FLI1 knockdown. (F) Colony formation assays and survival fraction curve analysis were employed to assess cell survival at 10-14 days after exposure to indicated IR dose. (G) Annexin V/PI double-staining assays were performed to evaluate the effects of FLI1 on apoptosis 48h after cells treated with or without IR. Data in C, E ,F and G are presented as mean ± SD (n=3). *p < 0.05, **p < 0.01, ***p < 0.001, ****p < 0.0001; ns, not significant (Student's t-test). Source data are provided as a Source Data file. Figure S3. Western blot of γ-H2AX protein levels in indicated NPC cells with or without IR. Figure S4. (A-B) Western blot (A) and RT-qPCR (B) analysis of TIE1 protein and mRNA level in FLI1 overexpression and knockdown NPC cells. (C) Dual-luciferase reporter assays were used to evaluate TIE1 promoter activity in NPC cells transiently transfected with control vector (VEC), FLI1 overexpression plasmid (FLI1), negative control siRNA (NC) and FLI1-specific siRNA (siFLI1). Figure S5. (A) Western blot analysis of TIE1, PI3K, p-PI3K, AKT, p-AKT (Thr308) and p-AKT (Ser473) in SUNE1 cells with FLI1 overexpression and 6-10B cells with FLI1 knockdown. (B) CNE1-VEC and CNE1-FLI1 cells were treated with IR, a PI3K inhibitor BKM120 (3μM) and an AKT inhibitor MK2206 (3μM). Western blot analysis was performed to detect the protein le [file 12967_2023_3986_MOESM1_ESM.pdf]
