# Supplementary material for: FLI1 regulates radiotherapy resistance in nasopharyngeal carcinoma through TIE1-mediated PI3K/AKT signaling pathway
Source: J Transl Med. 2023 Feb 22;21:134. doi: 10.1186/s12967-023-03986-y (PMC9945741; doi:10.1186/s12967-023-03986-y)
Supplement: Supplementary file 2 — Additional file 2: Table S1. Correlation between FLI1 and clinical characteristics in NPC patients. Table S2. Multivariate analysis of prognostic factors for OS in NPC patients. Table S3. Multivariate analysis of prognostic factors for LRFS in NPC patients. Table S4. Multivariate analysis of prognostic factors for PFS in NPC patients. Table S5. List of primers used in this study. Table S6. List of antibodies used in this study. [file 12967_2023_3986_MOESM2_ESM.pdf]

**Table S1. Correlation between FLI1 and clinical characteristics in NPC patients.**

| Variable | No.of patients | FLI1 | | $X^2$ | *P* Value |
|---|---|---|---|---|---|
| | | Low | High | | |
| Age, years | | | | 1.045 | 0.307 |
| <60 | 109 | 36 (33.0%) | 73 (67.0%) | | |
| ⩾60 | 41 | 10 (24.4%) | 31 (75.6%) | | |
| Gender | | | | 0.071 | 0.790 |
| Male | 112 | 35 (31.3%) | 77 (68.8%) | | |
| Female | 38 | 11 (28.9%) | 27 (71.1%) | | |
| AJCC stage | | | | 9.836 | 0.019 |
| Stage I | 17 | 8 (47.1%) | 9 (52.9%) | | |
| Stage II | 71 | 27 (38.0%) | 44 (62.0%) | | |
| Stage III | 42 | 6 (14.3%) | 36 (85.7%) | | |
| Stage IV | 20 | 5 (25.0%) | 15 (75.0%) | | |

**Table S2. Multivariate analysis of prognostic factors for OS in NPC patients.**

| Variable | HR (95% CI) | P value |
|---|---|---|
| Age, years | | <0.0001* |
| <60 | 1.00 (Reference) | |
| ⩾60 | 4.411 (2.472-7.872) | |
| Gender | | 0.762 |
| Female | 1.00 (Reference) | |
| Male | 0.893 (0.429-1.860) | |
| AJCC stage | | <0.0001* |
| Stage I | 1.00 (Reference) | |
| Stage II | 2.129 (0.275-16.470) | |
| Stage III | 7.464 (0.990-56.290) | |
| Stage IV | 12.941 (1.663-100.712) | |
| FLI1-TIE1 level * | | 0.029* |
| Low | 1.00 (Reference) | |
| High | 1.933 (1.071-3.487) | |

* FLI1-TIE1 level is defined as high when both FLI1 and TIE1 levels were high, and low when either FLI1 or TIE1 level was low.

**Table S3. Multivariate analysis of prognostic factors for LRFS in NPC patients.**

| Variable | HR (95% CI) | P value |
|---|---|---|
| Age, years | | 0.230 |
| <60 | 1.00 (Reference) | |
| ⩾60 | 1.383 (0.815-2.350) | |
| Gender | | 0.213 |
| Female | 1.00 (Reference) | |
| Male | 0.678 (0.368-1.249) | |
| AJCC stage | | <0.0001* |
| Stage I | 1.00 (Reference) | |
| Stage II | 7.963 (1.076-58.945) | |
| Stage III | 10.370 (1.387-77.509) | |
| Stage IV | 31.354 (4.121-238.564) | |
| FLI1-TIE1 level * | | 0.001* |
| Low | 1.00 (Reference) | |
| High | 2.223 (1.359-3.637) | |

* FLI1-TIE1 level is defined as high when both FLI1 and TIE1 levels were high, and low when either FLI1 or TIE1 level was low.

**Table S4. Multivariate analysis of prognostic factors for PFS in NPC patients.**

| Variable | HR (95% CI) | P value |
|---|---|---|
| Age, years | | 0.161 |
| <60 | 1.00 (Reference) | |
| ⩾60 | 1.463 (0.860-2.489) | |
| Gender | | 0.212 |
| Female | 1.00 (Reference) | |
| Male | 0.678 (0.369-1.248) | |
| AJCC stage | | <0.0001* |
| Stage I | 1.00 (Reference) | |
| Stage II | 7.815 (1.055-57.873) | |
| Stage III | 10.275 (1.375-76.804) | |
| Stage IV | 32.688 (4.302-248.375) | |
| FLI1-TIE1 level * | | 0.002* |
| Low | 1.00 (Reference) | |
| High | 2.137 (1.308-3.494) | |

* FLI1-TIE1 level is defined as high when both FLI1 and TIE1 levels were high, and low when either FLI1 or TIE1 level was low.

**Table S5. List of primers used in this study.**

| Gene | Sequence (5'to 3') |
|---|---|
| shRNA sequence | |
| shRNA-FLI1-F | CCGGGCTATATGGACGAGAAGAACTCGAGTTCTTCTCGTC CATATAGCTTTTTG |
| shRNA-FLI1-R | AATTCAAAAAGCTATATGGACGAGAAGAACTCGAGTTCT TCTCGTCCATATAGC |
| siRNA sequences | |
| siRNA-FLI1 | GCUAUAUGGACGAGAAGAATT |
| siRNA-TIE1 | GCAACGGAUCCUACUUCUATT |
| Real time RT-PCR primers | |
| GAPDH-F | GTCTCCTCTGACTTCAACAGCG |
| GAPDH-R | ACCACCCTGTTGCTGTAGCCAA |
| FLI1-F | ACGGAAGTGCTGTTGTCACACC |
| FLI1-R | CAAGCTCCTCTTCTGACTGAGTC |
| TIE1-F | ATGGCTGCTCTTGTGGATCTGG |
| TIE1-R | CGGTCACAAGTGCCACCATTCT |
| TIE1 promoter primers | |
| -1735 to +25 of the promoter region | |
| Forward | TTTCTCTATCGATAGGTACCacctgtgccaatatctgtgtca |
| Reverse | GATCGCAGATCTCGAGCTGCTCAGCCTGTGTTG |
| -1735 to -1507 of the promoter region | |
| Forward | TTTCTCTATCGATAGGTACCacctgtgccaatatctgtgtca |
| Reverse | GATCGCAGATCTCGAGagtggtggatgccttacacttca |

**Table S6. List of antibodies used in this study.**

| Antibody | Company | Catalog no. | Dilution |
|---|---|---|---|
| **Western blot** | | | |
| Anti-FLI1 Antibody, Rabbit Monoclonal | Abcam | ab133485 | 1:1000 |
| TIE1 (D2K2T), Rabbit mAb | CST | 23111S | 1:1000 |
| Cleaved Caspase-3 (Asp175) Antibody, Rabbit | CST | 9661S | 1:1000 |
| Anti-gamma H2A.X (phospho S139) Antibody, Mouse Monoclonal | Abcam | ab26350 | 1:1000 |
| PI3 Kinase p85 Antibody, Rabbit | CST | 4292S | 1:1000 |
| Anti-PI 3 Kinase p85 alpha (phospho Y607) Antibody, Rabbit Polyclonal | Abcam | ab182651 | 1:1000 |
| Akt (pan) (11E7), Rabbit mAb | CST | 4685S | 1:1000 |
| Phospho-Akt (Ser473) (D9E) XP® , Rabbit Monoclonal | CST | 4060S | 1:2000 |
| Phospho-Akt (Thr308) (D25E6) XP®, Rabbit Monoclonal | CST | 13038S | 1:1000 |
| Phospho-ATR (Ser428) Antibody, Rabbit | CST | 2853T | 1:1000 |
| Phospho-ATM (Ser1981) (D25E5), Rabbit mAb | CST | 13050S | 1:1000 |
| Phospho-Chk1 (Ser345) (133D3), Rabbit Monoclonal | CST | 2348T | 1:1000 |
| Phospho-Chk2 (Thr68) (C13C1), Rabbit Monoclonal | CST | 2197T | 1:1000 |
| GAPDH Antibody, Rabbit Polyclonal | Proteintech | 10494-1-AP | 1:5000 |
| HRP-conjugated Affinipure Goat Anti-Mouse IgG(H+L) | Proteintech | SA00001-1 | 1:10000 |
| HRP-conjugated Affinipure Goat Anti-Rabbit IgG(H+L) | Proteintech | SA00001-2 | 1:10000 |
| **Immunofluorescence** | | | |
| Anti-gamma H2A.X (phospho S139) Antibody, Mouse Monoclonal | Abcam | ab26350 | 1:200 |
| Cy3 Goat Anti-Mouse IgG (H+L) | Abclonal | AS008 | 1:200 |
| **Immunohistochemistry** | | | |
| Anti-FLI1 Antibody, Rabbit Monoclonal | Abcam | ab133485 | 1:400 |
| Anti-TIE1 antibody, Rabbit Polyclonal | Bioss | bs-1334R | 1:200 |
| Cleaved Caspase-3 (Asp175) Antibody, Rabbit | CST | 9661S | 1:400 |
| **ChIP** | | | |
| DYKDDDDK tag antibody (Binds to FLAG® tag epitope), Rabbit Polyclonal | Proteintech | 20543-1-AP | 2ug |
